# Singular Influence of the Aurora Borealis

**Published:** 1851-11

**Authors:** R. S. Miller

**Affiliations:** Telegrapher of Chicago


ARTICLE II.

*Singular Influence of the Aurora Borealis.* By R. S. MILLER, Esq., Telegrapher of Chicago.

The origin of that singular and beautiful Phenomenon, the "Aurora Borealis," (more familiarly termed the "Northern Lights") seems yet a mystery not only to the millions, but also to those who have given it a patient and careful investigation.

The different theories which have been advanced from time to time are now almost entirely abandoned, and the apparent want of sufficient data upon which to found anything plausable, has in a great measure checked the progress of inquiry. Some years since it was accidentally discovered that the polarity of the Magnetic needle suffered a material change during the presence of the "Lights." This gave a new impetus to investigation, but further than the simple fact itself, nothing new has been developed.

That it is an Electrical Phenomenon, is the only theory that prevails at the present day. It has met the support of Herschel and other distinguished Astronomers whose opinions are entitled to no little consideration.

During the very striking and brilliant exhibition of this Phenomenon on the night of the 30th ult., a new feature occurred under the immediate observation of the writer, which may be of interest to the scientific and furnish additional evidence in favor of the theory above mentioned.

On the night alluded to, the different Lines of Telegraph were affected in a singular manner, and to such an extent as to compel them to suspend operations for several hours. The derangement (as indicated by the Electro Magnets) at first appeared similar to that which would be expected to result from the crossing or contact of the wires of different Lines in the immediate vicinity of powerful batteries. An examination served to show that the difficulty could not proceed from such a cause. At one moment it was impossible (by the use of the usual tests) to detect the presence of the slightest current of magnetism on the wires. It would then gradually manifest itself and steadily increase for perhaps two minutes, at which time its intensity would be extraordinary; the Magnets exerting an attractive force equal to five or six pounds; (it is usually about one ounce.) It would then gradually diminish and finally disappear.

These alternate effects were regular, and the Magnets connected with the different Lines extending in different directions acted in perfect concert; thereby showing that the derangement was not confined to any particular locality; a fact more fully proven when it was ascertained that the same effects were being abserved in St. Louis, Cincinnati, Milwaukee and other stations eastward.

By some, these effects might have been attributed to electricity; but it would have been a forced conclusion. A perfect equilibrium appeared to prevail everywhere, and none of the usual indications of the presence of that agency were observed.

It would be tedious to enumerate all the experiments made on the occasion. Suffice it tor the present to say, that the result has convinced the writer that the derangement was occasioned by " *Magnetic Electricity*, " but where or how it was developed, or whether it be a cause or an effect of the Phenomenon in question is left for others to determine.

